# Supplementary figures and images for: Colour preferences of UK garden birds at supplementary seed feeders
Source: PLoS One. 2017 Feb 17;12(2):e0172422. doi: 10.1371/journal.pone.0172422 (PMC5315500; doi:10.1371/journal.pone.0172422)

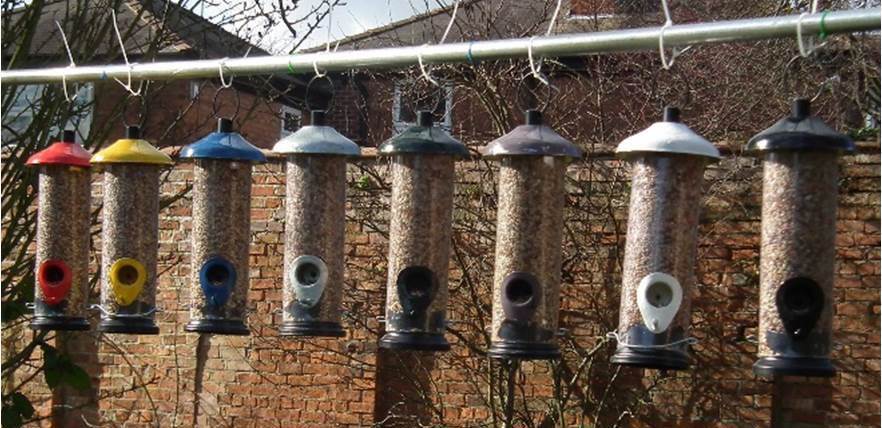

Supplement: S1 Fig — An example array of filled birdfeeders ready for observations in the field. The colour order (from left to right) is: red, yellow, blue, silver, green, purple, white, black. Colour order was randomised between trials. (JPG) [file pone.0172422.s001.jpg]

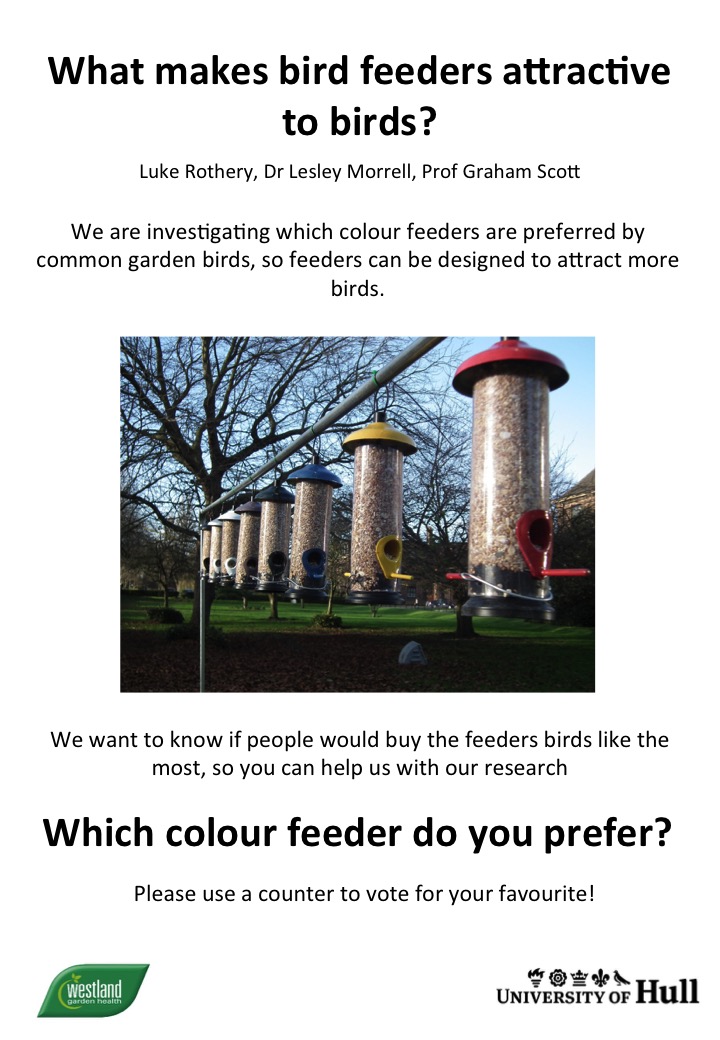

Supplement: S2 Fig — A copy of the poster explaining the project, as displayed at the Science Festival and in the garden centre. (JPG) [file pone.0172422.s002.jpg]
