# Supplementary material for: Colour preferences of UK garden birds at supplementary seed feeders
Source: PLoS One. 2017 Feb 17;12(2):e0172422. doi: 10.1371/journal.pone.0172422 (PMC5315500; doi:10.1371/journal.pone.0172422)
Supplement: S1 Table — The cells above the diagonal show the z- and p-values, while the estimate ± standard error is below the diagonal. Significant p-values are highlighted in bold. (PDF) [file pone.0172422.s003.pdf]

S1 Table: Pairwise comparisons of visits to feeders by blue tits. The cells above the diagonal show the z- and p-values, while the estimate ± standard error is below the diagonal. Significant p-values are highlighted in bold.

	Red	Yellow	Green	Blue	Purple	White	Silver	Black
Red	-	z = -0.334<br>p = 0.862	z = -2.132<br>p = 0.084	z = -2.521<br>**p = 0.041**	z = -2.668<br>**p = 0.035**	z = 1.810<br>p = 0.164	z = 3.153<br>**p = 0.023**	z = -2.668<br>**p = 0.035**
Yellow	-0.047±0.141	-	z =-2.466<br>**p = 0.042**	z = -2.855<br>**p = 0.024**	z = -2.922<br>**p = 0.024**	z = -2.145<br>p = 0.090	z = 3.485<br>**p = 0.013**	z = -3.001<br>**p = 0.025**
Green	-0.289±0.136	-0.336±0.136	-	z = -0.389<br>p = 0.849	z = 0.457<br>p = 0.824	z = -0.326<br>p = 0.833	z = 1.028<br>p = 0.608	z = -0.535<br>p = 0.829
Blue	-0.340±0.135	-0.387±0.135	-0.050±0.130	-	z = 0.069<br>p = 0.945	z = -0.716<br>p = 0.780	z = 0.642<br>p = 0.810	z = -0.147<br>p = 0.951
Purple	0.348±0.135	-0.396±0.135	0.059±0.130	0.009±0.129	-	z = -0.785<br>p = 0. 757	z = 0.5573<br>p = 0.835	z = -0.078<br>p = 0.972
White	0.246±0.136	-0.293±0.137	-0.043±0.131	-0.093±0.130	-0.102±0.130	-	z = -1.356<br>p = 0.377	z = -0.864<br>p = 0.724
Silver	0.422±0.134	-0.469±0.135	0.133±0.130	0.082±0.128	0.073±0.128	-0.176±0.129	-	z = 0.495<br>p = 0.827
Black	-0.358±0.134	-0.405±0.135	-0.069±0.129	-0.019±0.129	-0.010±0.128	-0.112±0.130	0.063±0.127	-
