# Supplementary material for: Colour preferences of UK garden birds at supplementary seed feeders
Source: PLoS One. 2017 Feb 17;12(2):e0172422. doi: 10.1371/journal.pone.0172422 (PMC5315500; doi:10.1371/journal.pone.0172422)
Supplement: S2 Table — The cells above the diagonal show the z- and p-values, while the estimate ± standard error is below the diagonal. Significant p-values are highlighted in bold. (PDF) [file pone.0172422.s004.pdf]

S2 Table: Pairwise comparisons of visits to feeders by great tits. The cells above the diagonal show the z- and p-values, while the estimate ± standard error is below the diagonal. Significant p-values are highlighted in bold.

	Red	Yellow	Green	Blue	Purple	White	Silver	Black
Red	-	z = -1.265<br>p = 0.501	z = -3.474<br>**p = 0.017**	z = -2.169<br>p = 0.157	z =-2.636<br>p = 0.088	z = 1.930<br>p = 0.285	z = 2.676<br>p = 0.084	z = -2.744<br>p = 0.068
Yellow	-0.260±0.206	-	z =-4.688<br>**p = 0.002**	z = -3.411<br>**p = 0.015**	z = -3.870<br>**p = 0.008**	z = -3.176<br>**p = 0.030**	z = -3.905<br>**p = 0.007**	z = -3.961<br>**p = 0.008**
Green	-0.658±0.189	0.919±0.195	-	z = 1.328<br>p = 0.552	z = -0.852<br>p = 0.739	z =-1.559<br>p = 0.371	z = -0.806<br>p = 0.760	z = 0.747<br>p = 0.739
Blue	-0.418±0.192	-0.678±0.199	0.240±0.181	-	z = 0.472<br>p = 0.902	z = -0.238<br>p = 0.872	z = 0.519<br>p = 0.849	z = -0.582<br>p = 0.824
Purple	-0.504±0.191	-0.765±0.198	-0.154±0.180	0.087±0.183	-	z = -0.709<br>p = 0.758	z = 0.047<br>p = 0.981	z = -0.108<br>p = 0.931
White	0.374±0.194	-0.634±0.200	-0.285±0.183	-0.044±0.186	-0.131±0.185	-	z = -0.755<br>p = 0.745	z = -0.818<br>p = 0.759
Silver	0.513±0.192	-0.773±0.198	-0.145±0.180	0.095±0.184	0.009±0.182	-0.139±0.185	-	z = -0.061<br>p = 0.949
Black	-0.524±0.191	-0.785±0.198	0.134±0.180	-0.106±0.183	-0.020±0.181	-0.151±0.184	-0.011±0.181	-
