# Supplementary material for: Colour preferences of UK garden birds at supplementary seed feeders
Source: PLoS One. 2017 Feb 17;12(2):e0172422. doi: 10.1371/journal.pone.0172422 (PMC5315500; doi:10.1371/journal.pone.0172422)
Supplement: S3 Table — The cells above the diagonal show the z- and p-values, while the estimate ± standard error is below the diagonal. Significant p-values are highlighted in bold. (PDF) [file pone.0172422.s005.pdf]

**S3 Table: Pairwise comparisons of visits to feeders by coal tits.** The cells above the diagonal show the z- and p-values, while the estimate ± standard error is below the diagonal. Significant p-values are highlighted in bold.

| | Red | Yellow | Green | Blue | Purple | White | Silver | Black |
|---|---|---|---|---|---|---|---|---|
| Red | - | z = -0.354<br>p = 0.837 | z = -0.518<br>p = 0.941 | z = 0.546<br>p = 0.814 | z = -1.652<br>p = 0.408 | z = -0.661<br>p = 0.728 | z = 2.929<br>p = 0.162 | z = -2.663<br>p = 0.150 |
| Yellow | -0.074±0.208 | - | z = -0.869<br>p = 0.766 | z = 0.191<br>p = 0.988 | z = -1.993<br>p = 0.203 | z = 0.305<br>p = 0.870 | z = -3.248<br>p = 0.097 | z = -2.996<br>p = 0.112 |
| Green | -0.104±0.200 | -0.177±0.204 | - | z = 1.063<br>p = 0.743 | z = 1.144<br>p = 0.496 | z = -1.179<br>p = 0.657 | z = 2.439<br>p = 0.188 | z = -2.162<br>p = 0.153 |
| Blue | 0.114±0.209 | 0.041±0.213 | 0.218±0.205 | - | z = 2.188<br>p = 0.194 | z = -0.166<br>p = 0.892 | z = 3.445<br>p = 0.085 | z = -3.178<br>p = 0.088 |
| Purple | -0.318±0.193 | -0.392±0.197 | 0.215±0.188 | 0.433±0.198 | - | z = -2.297<br>p = 0.165 | z = 1.307<br>p = 0.731 | z = -1.040<br>p = 0.679 |
| White | -0.139±0.210 | 0.065±0.214 | -0.243±0.206 | -0.225±0.215 | -0.457±0.199 | - | z = -3.559<br>p = 0.112 | z = -3.277<br>p = 0.151 |
| Silver | 0.545±0.186 | -0.619±0.190 | 0.442±0.181 | 0.659±0.191 | 0.227±0.174 | -0.684±0.192 | - | z = 0.258<br>p = 0.925 |
| Black | -0.501±1.888 | -0.575±0.192 | -0.398±0.184 | -0.616±0.194 | -0.183±0.176 | -0.640±0.195 | 0.044±0.169 | - |
