# Supplementary material for: Colour preferences of UK garden birds at supplementary seed feeders
Source: PLoS One. 2017 Feb 17;12(2):e0172422. doi: 10.1371/journal.pone.0172422 (PMC5315500; doi:10.1371/journal.pone.0172422)
Supplement: S4 Table — The cells above the diagonal show the z- and p-values, while the estimate ± standard error is below the diagonal. Significant p-values are highlighted in bold. (PDF) [file pone.0172422.s006.pdf]

S4 Table: Pairwise comparisons of visits to feeders by house sparrows. The cells above the diagonal show the z- and p-values, while the estimate ± standard error is below the diagonal. Significant p-values are highlighted in bold.

	Red	Yellow	Green	Blue	Purple	White	Silver	Black
Red	-	z = -2.811<br>**p = 0.009**	z = -4.822<br>**p < 0.001**	z = -4.440<br>**p < 0.001**	z = -1.691<br>p = 0.127	z = 1.212<br>p = 0.263	z = 3.117<br>**p = 0.004**	z = -4.383<br>**p < 0.001**
Yellow	-0.718±0.256	-	z = 6.959<br>**p < 0.001**	z = -6.649<br>**p < 0.001**	z = -4.324<br>**p < 0.001**	z = -3.909<br>**p < 0.001**	z = -5.550<br>**p < 0.001**	z = -6.600<br>**p < 0.001**
Green	-0.899±0.186	-0.162±0.232	-	z = 0.422<br>p = 0.698	z = -3.243<br>**p = 0.003**	z = -3.720<br>**p = 0.001**	z = -1.823<br>p = 0.101	z = 0.470<br>p = 0.688
Blue	-0.833±0.188	-1.552±0.233	0.065±0.155	-	z = -2.838<br>**p = 0.008**	z = -3.319<br>**p = 0.002**	z = -1.406<br>p = 0.203	z = 0.048<br>p = 0.961
Purple	-0.342±0.202	-1.060±0.245	-0.557±0.172	-0.492±0.173	-	z = -0.488<br>p = 0.701	z = 1.459<br>p = 0.193	z = -2.785<br>**p = 0.009**
White	0.248±0.205	-0.966±0.247	-0.650±0.175	-0.585±0.176	-0.094±0.192	-	z = -1.947<br>p = 0.080	z = -3.263<br>**p = 0.003**
Silver	0.604±0.194	-1.322±0.238	-0.295±0.162	-0.230±0.163	0.262±0.180	-0.356±0.183	-	z = -1.354<br>p = 0.214
Black	-0.825±0.188	-1.544±0.234	0.072±0.155	0.008±0.157	-0.484±0.174	-0.578±0.177	-0.222±0.164	-
