# Supplementary material for: Colour preferences of UK garden birds at supplementary seed feeders
Source: PLoS One. 2017 Feb 17;12(2):e0172422. doi: 10.1371/journal.pone.0172422 (PMC5315500; doi:10.1371/journal.pone.0172422)
Supplement: S5 Table — The cells above the diagonal show the z- and p-values, while the estimate ± standard error is below the diagonal. Significant p-values are highlighted in bold. (PDF) [file pone.0172422.s007.pdf]

S5 Table: Pairwise comparisons of visits to feeders by robins. The cells above the diagonal show the z- and p-values, while the estimate ± standard error is below the diagonal. Significant p-values are highlighted in bold.

	Red	Yellow	Green	Blue	Purple	White	Silver	Black
Red	-	z = 0.432<br>p = 0.741	z = -1.502<br>p = 0.266	z = -0.969<br>p = 0.489	z = 1.279<br>p = 0.436	z = -0.610<br>p = 0.592	z = 0.891<br>p = 0.578	z = -2.725<br>p = 0.121
Yellow	0.135±0.311	-	z = -1.116<br>p = 0.385	z = -0.562<br>p = 0.611	z = 1.584<br>p = 0.312	z = 1.127<br>p = 0.517	z = -0.138<br>p = 0.753	z = 02.387<br>p = 0.161
Green	-0.446±0.297	-0.324±0.290	-	z = 0.784<br>p = 0.600	z = -2.597<br>p = 0.059	z = -1.946<br>p = 0.128	z = -0.557<br>p = 0.580	z = -1.053<br>p = 0.631
Blue	-0.293±0.302	-0.166±0.295	0.218±0.278	-	z = -2.077<br>p = 0.153	z = -1.561<br>p = 0.288	z = -0.117<br>p = 0.832	z = -1.415<br>p = 0.426
Purple	0.447±0.349	0.545±0.344	-0.845±0.325	-0.684±0.329	-	z = 0.679<br>p = 0.581	z = 2.141<br>p = 0.152	z = -3.693<br>**p = 0.018**
White	-0.202±0.331	0.369±0.328	-0.599±0.308	-0.492±0.315	0.245±0.361	-	z = -1.205<br>p = 0.355	z = -3.251<br>**p = 0.049**
Silver	0.272±0.306	-0.041±0.299	-0.157±0.282	-0.034±0.289	0.723±0.338	-0.385±0.320	-	z = -1.745<br>p = 0.277
Black	-0.784±0.288	-0.671±0.281	-0.275±0.261	-0.377±0.266	-1.169±0.317	-0.980±0.302	-0.473±0.271	-
